# A clinical decision aid for patients with suspected midfacial and mandibular fractures (the REDUCTION-I study): a prospective multicentre cohort study

**DOI:** 10.1007/s00068-022-01968-1

**Published:** 2022-04-16

**Authors:** Romke Rozema, Mostafa El Moumni, Gysbert T. de Vries, Frederik K. L. Spijkervet, René Verbeek, Jurrijn Y. J. Kleinbergen, Bas W. J. Bens, Michiel H. J. Doff, Baucke van Minnen, the REDUCTION study group

**Affiliations:** 1 Department of Oral and Maxillofacial Surgery, University Medical Center Groningen, University of Groningen, Hanzeplein 1, 9700 RB Groningen, The Netherlands; 2 Department of Trauma Surgery, University Medical Center Groningen, University of Groningen, Groningen, The Netherlands; 3 Department of Emergency Medicine, Isala Hospital, Zwolle, The Netherlands; 4 Department of Emergency Medicine, Nij Smellinghe Hospital, Drachten, The Netherlands; 5 Department of Oral and Maxillofacial Surgery, Isala Hospital, Zwolle, The Netherlands; 6 Department of Oral and Maxillofacial Surgery, Nij Smellinghe Hospital, Drachten, The Netherlands; 7 Department of Emergency Medicine, University Medical Center Groningen, University of Groningen, Groningen, The Netherlands

**Keywords:** Maxillofacial fractures, Physical examination findings, Diagnostic accuracy, Sensitivity and specificity, Computed tomography, Cone-beam computed tomography, Clinical decision aid

## Abstract

**Purpose:**

To assess physical examination findings related to maxillofacial trauma to identify patients at risk of midfacial and mandibular fractures and then to construct a clinical decision aid to rule out the presence of midfacial and mandibular fractures in emergency department patients.

**Methods:**

We performed a prospective multicentre cohort study in four hospitals in the Netherlands, including consecutive patients with maxillofacial trauma. Each patient received a standardized physical examination consisting of 15 and 14 findings for midfacial and mandibular traumas, respectively. Consequently, clinical decision aids were constructed with the focus being on ruling out the presence of midfacial and mandibular fractures, and diagnostic accuracy was calculated.

**Results:**

A total of 993 consecutive patients were identified of whom 766 and 280 patients were suspected of midfacial and mandibular fractures, respectively. Midfacial fractures were diagnosed in 339 patients (44.3%), whereas mandibular fractures were observed in 66 patients (23.6%). The decision aid for midfacial trauma consisting of peri-orbital hematoma, epistaxis, ocular movement limitation, infra-orbital nerve paresthesia, palpable step-off and tooth mobility or avulsion, produced a sensitivity of 89.7 (86.0–92.5), a specificity of 42.6 (38.0–47.4), and a negative predictive value of 83.9% (78.4–88.2). The decision aid for mandibular trauma consisting of the angular compression test, axial chin pressure test, objective malocclusion, tooth mobility or avulsion and the tongue blade bite test resulted in a sensitivity of 98.5 (91.9–99.7), a specificity of 34.6 (28.5–41.2), and a negative predictive value of 98.7% (92.8–99.8).

**Conclusion:**

The constructed clinical decision aids for maxillofacial trauma may aid in stratifying patients suspected for midfacial and mandibular fractures to reduce unnecessary diagnostic imaging.

**Clinical Trial Registration:**

The study was registered at ClinicalTrials.gov with the identifier NCT03314480.

**Supplementary Information:**

The online version contains supplementary material available at 10.1007/s00068-022-01968-1.

## Introduction

Maxillofacial injuries comprise a substantial part of today’s emergency department visits. Computed tomography (CT) has been widely accepted as the routine imaging modality of choice for the diagnosis of these injuries. In the past decades, the increased use of CT has raised concerns regarding radiation dose associated risks, such as the carcinogenic potential [1]. Thus, clinical decisions aids were proposed to reduce unnecessary diagnostic imaging and associated health care costs.

The Wisconsin criteria were suggested as a clinical decision aid for midfacial and mandibular fractures [2]. Attempts to validate these criteria were unsuccessful in other studies [3–5]. A variety of studies published risk scores and clinical decision aids specifically for orbital fractures [6–9]. Separate clinical decision aids were also proposed for zygoma, orbital floor, nasal and mandibular fractures [4, 10, 11]. However, these decision aids have important limitations. First, a decision aid for specific fracture types is only useful for a selection of the maxillofacial trauma population. Second, combining midfacial and mandibular fractures as outcome does not allow for separate decision making for patients with isolated midfacial or mandibular trauma. Third, most studies collected data in single-centers resulting in geographic and demographic biases.

To our belief, a clinical decision aid for maxillofacial trauma should be straightforward and reproducible for all emergency department workers, including emergency physicians and specialized trauma surgeons. Moreover, a clinical decision aid should be applicable to both isolated and multitrauma patients. In today’s emergency care, diagnostic imaging is routinely considered in case of signs related to maxillofacial trauma. Therefore, it would be especially useful for identifying patients with a low risk of maxillofacial fractures thus reducing unnecessary imaging and, subsequently, in lowering radiation exposure and associated health care costs. In addition, we believe it would optimize the workflow of emergency department visits for these specific patients. We, therefore, initiated a prospective multicenter so called REDUCTION-I study (*RED*ucing *U*nnecessary *C*omputed *T*omography *I*n *M*axill*O*facial I*N*jury). The aim of this study was twofold. First, to assess the diagnostic accuracy of physical examination findings for patients with clinically suspected midfacial or mandibular trauma. Second, to construct a clinical decision aid with the focus being on ruling out the presence of midfacial and mandibular fractures in emergency department patients.

## Materials and methods

### Study design and ethical approval

A prospective multicenter observational cohort study was conducted of all patients admitted with a midfacial or mandibular trauma. The Medical Ethical Committee of the University Medical Center Groningen confirmed that the Medical Research Involving Human Subjects Act does not apply (METc code 2017/249) and local feasibility was approved for the Isala hospitals (METC171208) and Nij Smellinghe hospital (MEC6383/JS/AB). The study was performed in compliance with the Declaration of Helsinki and the FEDERA (Foundation Federation of Dutch Medical Scientific Societies) code of conduct. The study was registered at ClinicalTrials.gov (NCT03314480) and reported according to the STARD guidelines (Standards for Reporting of Diagnostic Accuracy Studies) and Methodologic Standards for Interpreting Clinical Decision Rules in Emergency Medicine [12, 13].

### Inclusion and exclusion criteria

All consecutive patients presenting with a midfacial or mandibular trauma at the emergency department of the University Medical Center Groningen (level I), Isala hospital Zwolle (Level I), Isala Diaconessenhuis hospital Meppel (level III) and Nij Smellinghe hospital Drachten (level III) between the period of May 2018 and October 2019 were included. Patients younger than 18 years and patients admitted a second time for maxillofacial trauma within the inclusion period were excluded. Patients were also excluded if the initial assessment was performed in another hospital or access to medical records was declined.

### Standardized assessment

All the eligible patients underwent a full physical examination consisting of 15 and 14 physical examination findings dedicated to the midfacial and mandibular region respectively. The physical examination was conducted by emergency physicians, surgeons or resident physicians of these professions. The process to standardize the physical examination was established using a tripartite strategy. First, each physician received an individual hands-on instruction on how to standardize each physical examination parameter. Second, we provided instructional videos on an open accessible online educational tool. Third, a pocket card was provided for bedside use, containing eligibility criteria and visualized physical examination findings. The findings were assessed during the primary or secondary assessment, and scored as absent, present or not assessable (supplementary Table S1). The findings were assessed without knowledge of the radiological imaging outcome, unless the emergent medical need of the patient required otherwise.

### Outcome and radiological imaging

The primary outcome was the presence of either a midfacial or mandibular fracture diagnosed with computed tomography (CT), cone beam computed tomography (CBCT) or panoramic orthopantomography (OPT). Midfacial fractures were defined as any fracture of the frontal sinus, orbital rim and walls, maxillary sinus, zygomaticomaxillary complex, nasoorbitoethmoid (NOE) complex, nasal bone, Le Fort I, II, III complex, and maxillary dentoalveolar complex. A mandibular fracture was defined as any fracture of the symphyseal, parasymphyseal, corpus, angular, ramus, coronoid and condylar process, and fractures of the mandibular dentoalveolar complex. CT datasets were assessed by radiologists, and CBCT and OPT were assessed by oral and maxillofacial surgeons. Radiological interpretations were performed blinded from the physical examination findings. Fracture classification was performed by a board certified oral and maxillofacial surgeon (BvM). Secondary outcomes were source of referral, mechanism of injury, age, reported alcohol use, state of consciousness in accordance with the Glasgow Coma Score, and status of intubation and sedation.

### Statistical analysis

The Statistical Package for the Social Sciences was used for the data analysis (IBM Corp. Released 2015. IBM SPSS Statistics for Windows, Version 23.0. Armonk, NY: IBM Corp.). Categorical variables were reported as frequencies and percentages. Normally distributed variables were reported as means and standard deviations, and variables with a skewed distribution were reported as median and inter quartile range. Normality was examined using Q–Q plots and tested using the Kolmogorov–Smirnov test. The diagnostic accuracy with corresponding 95% confidence intervals was calculated for the individual physical examination findings considering absent and present findings.

Principle component analysis (PCA) was used to construct a clinical decision aid for midfacial and mandibular traumas separately, with the focus being on identifying patients with a low fracture risk. The PCA analysis was performed with subsequent promax rotation and Kaiser normalization, and used to identify the underlying structure among physical examination findings. The Barlett’s test of sphericity and the Kaiser–Meyer–Olkin measure of sampling adequacy were conducted to test whether the variables were uncorrelated in the correlation matrix. Factors with Eigenvalues greater than one were initially retained. The factor loadings and clinical considerations of two board certified oral and maxillofacial surgeons (MD and BvM) were perused to identify the best combination of clinical physical examination findings to predict the presence of midfacial or mandibular fractures. Contingency tables were constructed with the absent findings being listed as ‘negative’, whereas the present not testable and missing findings were listed as a ‘positive’ findings. The diagnostic accuracy outcomes included: prevalence, pre-test probability, sensitivity, specificity, positive predictive value (PPV), negative predictive value (NPV), positive likelihood ratio (LR +) and negative likelihood ratio (LR – ).

## Results

### Patient identification

**Fig. 1:**
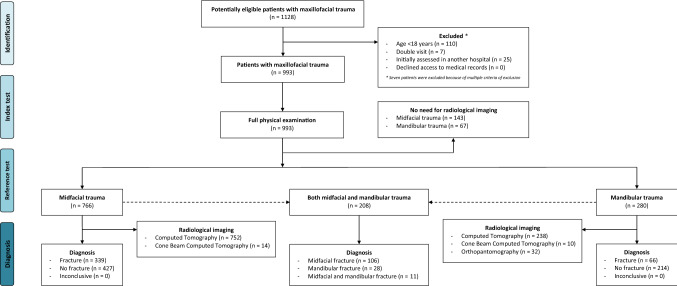
Flowchart of study patients

A total of 1128 consecutive patients with clinically suspected maxillofacial fracture(s) were screened in the 4 participating hospitals of whom 135 (12.0%) were excluded (Fig. 1). Seven patients were excluded because of multiple criteria of exclusion. Among the remaining 993 patients, 766 were suspected of midfacial fractures, 280 of mandibular fractures. Of the total population, 208 patients were suspected for both midfacial and mandibular fractures. Patient characteristics are summarized in Table 1.

**Table 1:** Patient characteristics

Total patients (*n*)	993
Hospital (*n* (%))	
University medical center Groningen	331 (33.3)
Isala hospital Zwolle	449 (45.2)
Isala Diaconessenhuis hospital Meppel	71 (7.2)
Nij Smellinghe hospital Drachten	142 (14.3)
Gender distribution (*n* (%))	
Male	522 (52.6)
Female	471 (47.4)
Age (years)	
Median	56
Interquartile range	41
Range	18–102
Source of referral (*n* (%))	
Ambulance	591 (59.5)
Air ambulance services	25 (2.5%)
General practitioner	254 (25.6%)
Dentist	8 (0.8%)
Self-referral	88 (8.9)
Other	27 (2.7%)
Mechanism of injury (*n* (%))	
Activities of daily living, home or private	312 (31.4)
Work	34 (3.4)
Traffic	443 (44.6)
Motor vehicles	10 (1.0)
Scooters and mopeds	37 (3.7)
Bicycles	334 (33.6)
Pedestrians	15 (1.5)
Sports	33 (3.3)
Violence	121 (12.2)
Fall from same level	4 (0.4)
Fall from high level	0 (0)
Suspected suicide attempt	3 (0.3)
Other	33 (3.3)
Not verifiable	10 (1.0)
Reported alcohol use (*n* (%))	226 (22.8)
GCS categories (*n* (%))	
Minor (14–15)	941 (94.8)
Moderate (9–13)	9 (0.9)
Severe (3–8)	33 (3.3)
Not reported	10 (1.0)
Status of intubation and/or sedation (*n* (%))	25 (2.5)

*GCS* Glasgow Coma Scale

### Radiological imaging

CT was obtained in 752 (98.2%) and CBCT in 14 (1.8%) midfacial trauma patients of whom fractures were diagnosed in 339 patients (44.3%). For mandibular trauma patients, CT was obtained in 238 (85.0%) patients, CBCT in 10 (3.6%) patients, and OPT in 32 (11.4%) patients. Mandibular fractures were diagnosed in 66 (23.6%) patients. Among the 208 patients with both midfacial and mandibular traumas, 106 (51.0%) of the patients only had midfacial fractures and 28 (13.5%) only had mandibular fractures. The remaining 11 (5.3%) patients had both midfacial and mandibular fractures.

### Physical examination findings for midfacial trauma patients

The diagnostic accuracy outcomes of the individual physical examination findings are summarized in Table 2. Swelling (81.1%), laceration (56.1%), peri-orbital hematoma (46.3%) and epistaxis (37.7%) were the most common physical examination findings for the midfacial trauma populations. Physical examination findings that were least common included ocular movement limitations (1.9%), diplopia (2.9%), and subjective (3.7%) and objective malocclusion (1.4%). High sensitivity was found for swelling (86.7%) and high specificity was found for facial depression (99.3), raccoon eyes (95.3), subconjunctival hemorrhage (95.9), ocular movement limitations (99.8), diplopia (98.3), infra-orbital nerve paresthesia (97.0), subjective (98.2) and objective malocclusion (98.6), tooth mobility or avulsion (96.5), palpable step-off (98.6) and maxillary mobility (96.1). A high PPV and corresponding LR + was found for facial depression (93.9/20.0), ocular movement limitations (92.3/16.3) and palpable step-off (91.2/13.6). For NPV of the individual physical findings was low, ranging from 57.5 to 70.6.

**Table 2:** Diagnostic accuracy of individual physical examination findings

Midface	Outcome				Statistics						
	Present (*n*)	Absent (*n*)	Not testable (*n*)	Missing data (*n*)	Prev. (CI)	Sens. (CI)	Spec. (CI)	PPV (CI)	NPV (CI)	LR + (CI)	LR – (CI)
Swelling	621	145	0	0	81.1 (78.1–83.7)	86.7 (82.7–89.9)	23.4 (19.7–27.7)	47.3 (43.4–51.3)	69.0 (61.0–75.9)	1.1 (1.1–1.2)	0.6 (0.4–0.8)
Laceration	430	336	0	0	56.1 (52.6–59.6)	59.0 (53.7–64.1)	46.1 (41.5–50.9)	46.5 (41.8–51.2)	58.6 (53.3–63.8)	1.1 (1.0–1.2)	0.9 (0.8–1.0)
Facial depression	49	693	21	3	6.6 (5.0–8.6)	14.3 (10.9–18.5)	99.3 (97.9–99.8)	93.9 (83.5–97.9)	60.2 (56.5–63.8)	20.0 (6.3–63.7)	0.9 (0.8–0.9)
Peri-orbital hematoma	355	411	0	0	46.3 (42.8–49.9)	58.7 (53.4–63.8)	63.5 (58.8–67.9)	56.1 (50.9–61.1)	65.9 (61.2–70.4)	1.6 (1.4–1.9)	0.7 (0.6–0.8)
Raccoon eyes	55	708	0	3	7.2 (5.6–9.3)	10.4 (7.6–14.1)	95.3 (92.9–96.9)	63.6 (50.4–75.1)	57.5 (53.8–61.1)	2.2 (1.3–3.8)	0.9 (0.9–1.0)
Epistaxis	285	470	9	2	37.7 (34.4–41.3)	58.4 (53.1–63.6)	78.5 (74.3–82.1)	68.1 (62.4–73.2)	70.6 (66.4–74.6)	2.7 (2.2–3.3)	0.5 (0.5–0.6)
Subconjunctival hemorrhage	69	656	36	5	9.5 (7.6–11.9)	16.6 (12.9–21.1)	95.9 (93.5–97.4)	75.4 (64.0–84.0)	60.1 (56.3–63.7)	4.0 (2.4–6.8)	0.9 (0.8–0.9)
Ocular movement limitation	13	686	65	2	1.9 (1.1–3.2)	4.1 (2.3–7.0)	99.8 (98.6–100.0)	92.3 (66.7–98.6)	58.6 (54.9–62.2)	16.3 (2.1–125.0)	1.0 (0.9–1.0)
Diplopia	20	676	68	2	2.9 (1.9–4.4)	4.4 (2.6–7.4)	98.3 (96.5–99.2)	65.0 (43.3–81.9)	58.6 (54.8–62.2)	2.6 (1.0–6.3)	1.0 (0.9–1.0)
Infra-orbital nerve paresthesia	62	635	66	3	8.9 (7.0–11.2)	17.1 (13.2–21.8)	97.0 (94.9–98.3)	80.6 (69.1–88.6)	61.7 (57.9–65.4)	5.7 (3.1–10.6)	0.9 (0.8–0.9)
Subjective malocclusion ^1^	13	342	183	1	3.7 (2.2–6.2)	6.3 (3.3–11.5)	98.1 (95.2–99.3)	69.2 (42.4–87.3)	60.5 (55.3–65.6)	3.3 (1.0–10.5)	1.0 (0.9–1.0)
Objective malocclusion ^1^	5	353	179	2	1.4 (0.6–3.2)	1.4 (0.4–5.1)	98.6 (96.1–99.5)	40.0 (11.8–76.9)	61.5 (56.4–66.4)	1.1 (0.2–6.3)	1.0 (1.0–1.0)
Tooth mobility or avulsion	48	707	8	3	6.4 (4.8–8.3)	10.0 (7.2–13.7)	96.5 (94.2–97.8)	68.8 (54.7–80.1)	57.9 (54.2–61.4)	2.8 (1.6–5.1)	0.9 (0.9–1.0)
Palpable step-off	68	663	29	6	9.3 (7.4–11.6)	19.6 (15.6–24.4)	98.6 (96.9–99.3)	91.2 (82.1–95.9)	61.7 (57.9–65.3)	13.6 (5.9–31.0)	0.8 (0.8–0.9)
Maxillary mobility	36	680	38	12	5.0 (3.7–6.9)	6.5 (4.3–9.8)	96.1 (93.7–97.6)	55.6 (39.6–70.5)	57.8 (54.0–61.5)	1.7 (0.9–3.2)	1.0 (0.9–1.0)
Mandible											
Swelling	105	175	0	0	37.5 (32.0–43.3)	75.8 (64.2–84.5)	74.3 (68.1–79.7)	47.6 (38.3–57.1)	90.9 (85.7–94.3)	2.9 (2.3–3.8)	0.3 (0.2–0.5)
Extra-oral laceration	101	179	0	0	36.1 (30.7–41.8)	54.5 (42.6–66.0)	69.6 (63.2–75.4)	35.6 (27.0–45.4)	83.2 (77.1–88.0)	1.8 (1.3–2.4)	0.7 (0.5–0.9)
Jaw movement pain	129	142	9	0	47.6 (41.7–53.5)	95.2 (86.7–98.3)	66.5 (59.9–72.6)	45.7 (37.4–54.3)	97.9 (94.0–99.3)	2.8 (2.3–3.5)	0.1 (0.0–0.2)
Mouth opening limitation	88	185	7	0	32.2 (27.0–38.0)	87.1 (76.6–93.3)	83.9 (78.3–88.2)	61.4 (50.9–70.9)	95.7 (91.7–97.8)	5.4 (3.9–7.5)	0.2 (0.1–0.3)
Inferior alveolar nerve paresthesia	8	253	17	2	3.1 (1.6–5.9)	6.7 (2.6–15.9)	98.0 (95.0–99.2)	50.0 (21.5–78.5)	77.9 (72.4–82.5)	3.4 (0.9–13.0)	1.0 (0.9–1.0)
Intra-oral hematoma	35	229	13	3	13.3 (9.7–17.9)	33.3 (22.5–46.3)	92.3 (87.8–95.2)	54.3 (38.2–69.5)	83.4 (78.0–87.7)	4.3 (2.4–7.8)	0.7 (0.6–0.9)
Intra-oral laceration	62	206	10	2	23.1 (18.5–28.5)	40.7 (29.1–53.4)	81.8 (76.0–86.5)	38.7 (27.6–51.2)	83.0 (77.3–87.5)	2.2 (1.5–3.4)	0.7 (0.6–0.9)
Palpable step-off	17	252	9	2	6.3 (4.0–9.9)	26.7 (17.1–39.0)	99.5 (97.3–99.9)	94.1 (73.0–99.0)	82.5 (77.4–86.7)	55.7 (7.5–411.7)	0.7 (0.6–0.9)
Tooth mobility or avulsion	18	256	5	1	6.6 (4.2–10.1)	17.5 (10.0–28.6)	96.7 (93.3–98.4)	61.1 (38.6–79.7)	79.7 (74.3–84.2)	5.3 (2.1–13.0)	0.9 (0.8–1.0)
Subjective malocclusion ^1^	31	27	7	0	55.0 (42.5–66.9)	81.8 (65.6–91.4)	77.8 (59.2–89.4)	81.8 (65.6–91.4)	77.8 (59.2–89.4)	3.7 (1.8–7.6)	0.2 (0.1–0.5)
Objective malocclusion ^1^	22	37	6	0	37.3 (26.1–50.0)	63.6 (46.6–77.8)	96.2 (81.1–99.3)	95.5 (78.2–99.2)	67.6 (51.5–80.4)	16.5 (2.4–115.0)	0.4 (0.2–0.6)
Angular compression test pain	96	171	12	1	36.0 (30.4–41.9)	85.0 (73.9–91.9)	78.3 (72.2–83.3)	53.1 (43.2–62.8)	94.7 (90.3–97.2)	3.9 (3.0–5.2)	0.2 (0.1–0.4)
Axial chin pressure pain	82	178	17	3	31.5 (26.2–37.4)	84.7 (73.5–91.8)	84.1 (78.4–88.5)	61.0 (50.2–70.8)	94.9 (90.7–97.3)	5.3 (3.8–7.4)	0.2 (0.1–0.3)
Tongue blade bite test	22	148	86	24	12.9 (8.7–18.8)	51.7 (34.4–68.6)	95.0 (90.1–97.6)	68.2 (47.3–83.6)	90.5 (84.7–94.3)	10.4 (4.7–23.3)	0.5 (0.3–0.7)

Abbreviations: *Prev* prevalence, *Sens* sensitivity, *Spec* specificity, *PPV* positive predictive value, *NPV* negative predictive value, LR + positive likelihood ratio, *LR – * negative likelihood ratio

^1^Excluding patients with both midfacial and mandibular traumas

### Physical examination findings for mandibular trauma patients

For mandibular trauma patients, jaw movement pain (47.6%) and subjective malocclusion (55.0%) were the most common physical examination findings, whereas inferior alveolar nerve paresthesia (3.1%), palpable step-off (6.3%) and tooth mobility or avulsion (6.6%) were less common. High sensitivity was found for jaw movement pain (95.2) and high specificity was found for inferior alveolar nerve paresthesia (98.0), intra-oral hematoma (92.3), palpable step-off (99.5), tooth mobility or avulsion (96.7), objective malocclusion (96.2) and the tongue blade bite test (95.0). A high PPV and LR + was found for palpable step-off (94.1/55.7) and objective malocclusion (95.5/16.5), whereas high NPV and low LR- was found for swelling (90.9/0.3), jaw movement pain (97.9/0.1), mouth opening limitations (95.7/0.2), the angular compression test (94.7/0.2), the axial chin pressure test (94.9/0.2) and the tongue blade bite test (90.5/0.5).

### Clinical decision aids

A clinical decision aid was constructed for midfacial and mandibular traumas based on the positive factor loadings and the findings that were considered to be clinically relevant. The decision aid for midfacial trauma consisted of: peri-orbital haematoma, epistaxis, ocular movement limitation, infra-orbital nerve paraesthesia, palpable step-off and tooth mobility or avulsion resulting in sensitivity of 89.7 (86.0–92.5), NPV of 83.9 (78.4–88.2) and a LR- of 0.2 (0.2–0.3) (Table 3). Thereby, a total of 182 (23.8%) truly negative patients were identified when all the physical examination findings were absent. The fracture types that were ruled out by the clinical decision aid included orbital fractures (*n* = 9), zygomaticomaxillary complex fractures (*n* = 15) and nasal bone fractures (*n* = 12) (Table 4). Regarding mandibular trauma patients, the decision aid consisted of the angular compression test, axial chin pressure test, objective malocclusion, tooth mobility or avulsion and the tongue blade bite test, resulting in a sensitivity of 98.5 (91.9–99.7), NPV of 98.7 (92.8–99.8) and a LR –  of 0.0 (0.0–0.3) (Table 3). A total of 74 (26.4%) truly negative patients were identified when all these physical examination findings were absent. The clinical decision aid did not rule out a symphyseal/parasymphyseal fracture (*n* = 1). The contingency tables for the individual physical examination findings and the clinical decision aids are presented in supplementary Table S2.

**Table 3:** Clinical decision aid for ruling out patients with midfacial and mandibular fractures

Clinical decision aid	Physical examination finding	Definition	Contingency table outcome		Cumulative diagnostic accuracy					
			TN (%)	FN (%)	Sens. (CI)	Spec. (CI)	PPV (CI)	NPV (CI)	LR + (CI)	LR- (CI)
Midfacial trauma	Peri-orbital hematoma	Any hematoma of the orbital or zygomaticomaxillary area that is not defined as swelling or raccoon eyes	271 (35.4)	140 (18.3)	58.7 (53.4–63.8)	63.5 (58.8–67.9)	56.1 (50.9–61.1)	65.9 (61.2–70.4)	1.6 (1.4–1.9)	0.7 (0.6–0.8)
	Epistaxis	A unilateral or bilateral active or past nosebleed	211 (27.5)	63 (8.2)	81.4 (76.9–85.2)	49.4 (44.7–54.1)	56.1 (51.7–60.4)	77.0 (71.7–81.6)	1.6 (1.4–1.8)	0.4 (0.3–0.5)
	Ocular movement limitation	Unilateral restricted gazing or limited eye movements in any direction	205 (26.8)	56 (7.3)	83.5 (79.2–87.1)	48.0 (43.3–52.7)	56.0 (51.7–60.3)	78.5 (73.2–83.1)	1.6 (1.4–1.8)	0.3 (0.3–0.4)
	Infra-orbital nerve paresthesia	Any numbness, change or loss of sensation of the infra-orbital nerve area of innervation	195 (25.5)	52 (6.8)	84.7 (80.4–88.1)	45.7 (41.0–50.4)	55.3 (51.0–59.5)	78.9 (73.4–83.6)	1.6 (1.4–1.7)	0.3 (0.3–0.4)
	Palpable step-off	The presence of a bony step-off found during palpation of the zygomatic arch, infra-orbital rim, supra and lateral orbital rim, nasal bridge and zygomaticoalveolar crest intra-orally	189 (24.7)	43 (5.6)	87.3 (83.3–90.4)	44.3 (39.6–49.0)	55.4 (51.2–59.6)	81.5 (76.0–85.9)	1.6 (1.4–1.7)	0.3 (0.2–0.4)
	Tooth mobility or avulsion	Mobility or avulsion of any maxillary tooth element	182 (23.8)	35 (4.6)	89.7 (86.0–92.5)	42.6 (38.0–47.4)	55.4 (51.2–59.5)	83.9 (78.4–88.2)	1.6 (1.4–1.7)	0.2 (0.2–0.3)
Mandible trauma	Angular compression test pain	Noteworthy presence of pain of the symphyseal or parasymphyseal region induced by simultaneous bilateral pressure of the mandibular angle	162 (57.9)	9 (3.2)	86.2 (75.7–92.5)	75.7 (69.5–81.0)	51.9 (42.5–61.0)	94.7 (90.3–97.2)	3.5 (2.7–4.6)	0.2 (0.1–0.3)
	Axial chin pressure test pain	Noteworthy unilateral or bilateral pain of the condylar or temporomandibular region induced by axial pressure on the chin	152 (54.3)	3 (1.1)	95.5 (87.5–98.4)	71.0 (64.6–76.7)	50.4 (41.8–59.0)	98.1 (94.5–99.3)	3.3 (2.7–4.1)	0.1 (0.0–0.2)
	Objective malocclusion	Objectively identified traumatic misalignment of the maxillary and mandibular dental arches	102 (36.4)	2 (0.7)	97.0 (89.6–99.2)	47.7 (41.1–54.3)	36.4 (29.6–43.7)	98.1 (93.3–99.5)	1.9 (1.6–2.1)	0.1 (0.0–0.3)
	Tooth mobility or avulsion	Mobility or avulsion of any mandibular tooth element	96 (34.3)	2 (0.7)	97.0 (89.6–99.2)	40.7 (34.3–47.3)	33.5 (27.2–40.5)	97.8 (92.2–99.4)	1.6 (1.5–1.8)	0.1 (0.0–0.3)
	Tongue blade bite test	The patient’s ability to maintain bilateral inter-maxillary fixation of a tongue depressor (1.6 mm) while it gets broken by rotation	74 (26.4)	1 (0.4)	98.5 (91.9–99.7)	34.6 (28.5–41.2)	31.7 (25.7–38.4)	98.7 (92.8–99.8)	1.5 (1.4–1.7)	0.0 (0.0–0.3)

Abbreviations: TN true negatives; FN false negatives; NPV negative predictive value; LR- negative likelihood ratio; Sens. Sensitivity

**Table 4:** Fracture outcomes

Clinical decision aid	False negatives (*n*)	True positives (*n*)	Total (*n*)
Midface	35	304	339
Frontal sinus	1	24	25
Orbital rim and walls	9	87	96
Maxillary sinus	4	26	30
Zygomaticomaxillary complex	15	119	134
Nasoorbitoethmoid complex	0	17	17
Nasal bone	12	114	126
Le Fort I	1	8	9
Le Fort II	1	7	8
Le Fort III	0	6	6
Dentoalveolar complex	1	14	15
Mandible	1	65	66
Symphyseal or parasymphyseal	1	23	24
Corpus	0	17	17
Angular	0	8	8
Ramus	0	7	7
Coronoid	0	4	4
Condylar process	0	44	44
Dentoalveolar complex	0	1	1

## Discussion

Emergency department workers are frequently faced with patients suspected of fractures of the maxillofacial region. Both the midfacial and mandibular regions are characterized by a set of distinctive physical examination findings that can be used to predict the likelihood of a fracture and, consequently, justify the need for radiological imaging. Clinical decision aids were constructed for this large prospective multicentre cohort study of emergency department patients with the aim to diagnose or rule out the presence of midfacial and mandibular fractures. We found that the clinical decision aid for midfacial trauma patients produced a sensitivity of 89.7 and NPV of 83.9, correctly identifying 23.8% of the patients who did not have a fracture. The aid for mandibular trauma patients gave a sensitivity of 98.5 and NPV of 98.7, thus identifying that 26.4% of the population did not have a fracture. Hence, the clinical decision aid can be used to reduce unnecessary use of radiological imaging, consequently reducing radiation exposure and associated health care costs for this population of patients.

Physical examination findings related to midfacial trauma are based on the distinctive and complex anatomy of the midface. Since facial depression, ocular movement limitations, infra-orbital nerve paraesthesia and palpable step-off individually had a high PPV (> 90) and LR + (> 10) means a midfacial fracture is very likely when one or more of these findings is present. Our clinical decision aid, consisting of peri-orbital haematoma, epistaxis, ocular movement limitation, infra-orbital nerve paraesthesia, palpable step-off and tooth mobility or avulsion, was unable to identify over 90% of the patients without fractures. Although 182 patients were correctly identified as not having a fracture, 35 patients were missed among whom orbital, zygomaticomaxillary complex and nasal fractures were most common. Clinical decision aids for midfacial trauma were also proposed by other authors. For example, the Wisconsin criteria uses a combination of bony step-off or instability, peri-orbital swelling or contusion, a Glasgow Coma Scale score of less than 14, malocclusion and tooth absence 2. The authors combined midfacial and mandibular fractures as an outcome. The presence of any of these findings resulted in a NPV of 87.8 and a sensitivity of 98.2, whereas the validation of these criteria by three studies resulted in a NPV of 81.3, 28.6 and 60, and a sensitivity of 97.4, 90.0 and 81.0, respectively. The other authors presented clinical decision aids for specific midfacial fracture types [8, 14]. A decision tool for orbital fractures was defined as any presence of subconjunctival hemorrhage, infra-orbital nerve paraesthesia, a change in position of the globe, reduced visual acuity or any two from peri-orbital hemorrhage, diplopia or limited eye movement, resulting in a NPV of 56.3 and a sensitivity of 80.0 8. Another study provided a decision tool for orbital floor fractures in the presence of any of the following: subconjunctival hemorrhage, infra-orbital nerve paraesthesia and ecchymosis or swelling, and resulted in a NPV and PPV of 92.3 and 74.2, respectively [14]. They also provided a decision aid for nasal and zygomaticomaxillary fractures, showing a NPV of 97.8 and 90.9. The clinical decision aids presented in their study were specifically constructed to rule out particular fracture types. However, from a clinical perspective, emergency department workers are blinded for the outcome of interest and, therefore, need to consider the full range of potential fracture types. In this present study, any midfacial fracture was chosen as an outcome to reflect the emergency department setting. Post hoc analysis can provide evidence of how the physical examination findings are related to these fracture types.

The distinctive mandibular trauma physical examination findings are related to the bilateral temporomandibular joint articulation that allows dental occlusion and articulation by mandibular motion. As individual findings, palpable step-off and objective malocclusion were found to have a particularly high PPV and LR + and so, therefore, strongly suggest the presence of a mandibular fracture. On the other hand, swelling, jaw movement pain, mouth opening limitation, the angular compression test, the axial chin pressure test and the tongue blade bite test gave a high NPV and LR – , indicating that these individual findings seems particularly useful for excluding the presence of mandibular fractures. This is in line with previous research which also found a high NPV for the tongue blade bite test [14–16]. We have successfully constructed a clinical decision aid for mandibular trauma patients resulting in a NPV of 98.7 and so identifying, retrospectively, that 26.4% of the patients had redundantly received radiological imaging. The clinical decision aid only missed one patient with a symphyseal fracture. Moreover, the cumulative diagnostic accuracy revealed that the clinical decision aid without the tongue blade bite test resulted in a NPV of 98.0 and so identified 34.3% of the patients correctly. There are no data available from previous research on the angular compression test and the axial chin pressure test. The utility of these tests seems particularly useful because of their generalizability and reproducibility for all emergency department workers. The diagnostic work-up for mandibular trauma patients is unique because OPT and posteroanterior mandibular radiographs can be used as primary diagnostic modality. However, this study provided evidence that CT is preferred because of the patients’ positioning, other diagnostic needs or medical treatment urgency in the setting of the emergency department.

Although the clinical decision aids in this study provide recommendations for ruling out midfacial and mandibular fractures, each patient should receive a standardized physical examination of the maxillofacial region. The presented findings and corresponding diagnostic accuracy can be used to stratify which patient at risk for maxillofacial fractures and subsequently require radiological imaging. Although, the prevalence varied in this study, each physical examination findings may have predictive value for specific fractures types. For example, ocular movement limitation and maxillary mobility were found in only 1.9% and 5.0% of the patients but may be beneficial in identifying orbital fractures and the more severe Le Fort type fractures, respectively. In addition, ophthalmologic findings including perception to light, color, form, visual acuity, pupillary reactions and fundus examination are not included in the present study but should be assessed for each patient to detect intrabulbar hemorrhage, retinal edema, detachment and optic nerve compression [17]. As each of these findings may identify specific conditions, they should be tested to the best possible, also in patients who are intubated or have an altered state of consciousness.

The methodological strengths of the study include the prospective multicenter study design, the large number of consecutive patients and the standardized physical examinations strategy for each patient. Furthermore, we included patients whose physical examination findings were stated as ‘not testable’. On using this approach, we were able to include patients who could not be assessed because of, for example, severe swelling with respect to ocular related findings, or the state of consciousness since interaction with the patients is necessary. The results of our study show that the physical examinations of both midfacial and mandibular trauma patients can lead to “not testable” findings, emphasizing the need to score this as an outcome. Previous studies did not report these specific outcomes. Most importantly, our approach to constructing the clinical decision aids makes them applicable to a full range of midfacial and mandibular trauma patients, regardless of age, mechanism of injury or trauma severity.

Limitations of our study include that the physical examinations were performed by various professions, each with different years of experience. However, from a clinical perspective, the diagnostic management of these patients is also conducted by a multidisciplinary team. Another limitation is that no validation was performed. Therefore, future research should focus on how these clinical decision aids safely can reduce unnecessary radiological imaging in a prospective cohort study with a new population of patients. Future research should also focus on how physical examination findings are related to midfacial or mandibular fractures that require immediate intervention. For example, orbital floor fractures are known for the potential entrapment of the inferior rectus muscle, causing ocular movement limitations that require surgical exploration and should, therefore, not to be missed.

In conclusion, the diagnostic accuracy of physical examination findings was identified for patients with suspected midfacial and mandibular fractures. The construction of a clinical decision aid resulted in a NPV of 83.9 for midfacial trauma patients and a NPV of 98.7 for mandibular trauma patients and may aid in stratifying patients suspected for fractures to reduce unnecessary diagnostic imaging.

## Standards of reporting

The study was reported according to the STARD guidelines (Standards for Reporting of Diagnostic Accuracy Studies) and Methodologic Standards for Interpreting Clinical Decision Rules in Emergency Medicine [12, 13].

## Supplementary Information

Below is the link to the electronic supplementary material.

Supplementary file1 (DOCX 23 KB)

Supplementary file2 (XLSX 292 KB)

## Data Availability

Included as supplementary material.
