# Supplementary material for: A clinical decision aid for patients with suspected midfacial and mandibular fractures (the REDUCTION-I study): a prospective multicentre cohort study
Source: Eur J Trauma Emerg Surg. 2022 Apr 16;48(5):4243–54. doi: 10.1007/s00068-022-01968-1 (PMC9532332; doi:10.1007/s00068-022-01968-1)
Supplement: Supplementary file 1 — Supplementary file1 (DOCX 23 KB) [file 68_2022_1968_MOESM1_ESM.docx]

| **Supplementary table S1 – Definition of the physical examination findings** | |
| --- | --- |
| **Midface** | |
| Swelling | Any swelling of the midfacial region. A “*yes*” was scored if there was swelling of the midfacial region. A “*no*” was scored if there was no swelling of the midfacial region. A “*not testable*” was scored if swelling of the midfacial region could not be assessed. |
| Laceration | Any (extra-oral) laceration of the midfacial skin. A “*yes*” was scored if there was any (extra-oral) laceration of the midfacial skin. A “*no*” was scored if there were no (extra-oral) laceration of the midfacial skin. A “*not testable*” was scored if laceration of the midfacial skin could not be assessed. |
| Facial depression | Unilateral flattening or depression of the malar eminence, cheek or zygomaticomaxillary complex. A “*yes”* was scored if there was unilateral facial depression. A “*no*” was scored if there was no unilateral facial depression. A “*not testable”* was scored if unilateral facial depression could not be assessed, for example in situations where physical examination was not possible due severe swelling or excessive pain during palpation. |
| Peri-orbital hematoma | Any hematoma localized within or around the orbital or zygomaticomaxillary area that is not defined as raccoon eyes. A “*yes*” was scored if there was a peri-orbital hematoma. A “*no*” was scored if there was no peri-orbital hematoma. A “*not testable*” was scored if the presence of a peri-orbital hematoma could not be assessed. |
| Raccoon eyes | Bilateral ecchymosis or hematoma localized within and around the upper and/or lower eyelids. A “*yes*” was scored for cases with raccoon eyes. A “*no*” was scored if raccoon eyes were not present. A “*not testable*” was scored if the presence of raccoon eyes could not be assessed. |
| Epistaxis | A unilateral or bilateral active or passed nosebleed. A “*yes*” was scored for cases with (past) epistaxis. A “*no*” was scored if there was no (past) epistaxis. A “*not testable*” was scored if the presence of epistaxis could not be assessed. |
| Subconjunctival hemorrhage | A sharply circumscribed bleeding or hemorrhage of the conjunctiva in one or both globes. A “*yes*” was scored if there was subconjunctival hemorrhage. A “*no*” was scored if there was no subconjunctival hemorrhage. A “*not testable*” was scored if the presence of a subconjunctival hemorrhage could not be assessed, for example where severe swelling or hematoma of the eye could not be visualized, even with active help. |
| Ocular movement limitation | Any restricted gazing or limitation of the eye movements in any direction. A “*yes*” was scored if there were ocular movement limitations. A “*no*” was scored if there were no ocular movement limitations. A “*not testable*” was scored if ocular movements could not be assessed, for example in case of severe swelling and/or hematoma or if patient’s state of consciousness hindered active instruction of the patient. |
| Diplopia | Double vision (a situation where the patients sees a single object in duplicate), either passive or induced during the assessment of the ocular movements. A “*yes*” was scored in case of diplopia. A “*no*” was scored if there was no diplopia. A “*not testable*” was scored if the presence of diplopia could not be assessed, for example when the patient’s state of consciousness hindered communication. |
| Infra-orbital nerve paresthesia | Any numbness, change or loss of sensation of the infraorbital nerve innervation area (e.g., the lower eyelid, nasal vestibule, part of the cheek, the upper lip, upper incisor, canine and premolars). A “*yes*” was scored if there was infra-orbital nerve paresthesia. A “*no*” was scored if there was no infra-orbital nerve paresthesia. A “*not testable*” was scored if the presence of infra-orbital nerve paresthesia could not be assessed, for example if the patient’s state of consciousness hindered communication. |
| Subjective malocclusion | Misalignment or incorrect relation between the teeth of the two dental arches as experienced by the patient that emerged after the trauma. A “*yes*” was scored if the patient experiences the presence of malocclusion. A “*no*” was scored if the patient did not experience malocclusion. A “*not testable*” was scored if subjective malocclusion could not be assessed, for example in a situation where the  patient’s state of consciousness hindered communication, or in edentulous patients. |
| Objective malocclusion | Traumatic misalignment or incorrect relation between the teeth of the two dental arches as objectively identified by the assessor during intra-oral examination assessment. A “*yes*” was scored in there was malocclusion. A “*no*” was scored if there was no malocclusion. A “*not testable*” was scored if the occlusion could not be assessed, for example if the patient had dentures, was edentulous or if the patient was intubated and sedated. |
| Tooth mobility or avulsion | Mobility or avulsion of any maxillary tooth elements (e.g., first (11-18) and second (21-28) quadrant tooth elements). A “*yes*” was scored in case of tooth mobility or avulsion. A “*no*” was scored if there was no tooth mobility or avulsion. A “*not testable*” was scored if the presence of tooth mobility or avulsion could not be assessed, for example if the patient could not be suitably instructed. |
| Palpable step-off | The presence of a bony step-off, step defect or discontinuity found when palpating any of the following midfacial anatomical landmarks: (1) zygomatic arch, (2) infra-orbital rim, (3) supra and lateral orbital rim, (4) nasal bridge and (5) zygomaticalveolar crest intra orally. A “*yes*” was scored if there was a palpable step-off. A “*no*” was scored if there was no palpable step-off. A “*not testable*” was scored if the presence of a palpable step-off could not be assessed, for example in case where swelling and/or hematoma hindered full palpable examination of the midface. |
| Maxillary mobility | Mobility of the complete alveolar process of the maxilla during active lateral or frontal palpation after fixation of the midface. A “*yes*” was scored in case of maxillary mobility. A “*no*” was scored if there was no maxillary mobility. A “*not testable*” was scored if maxillary mobility could not be assessed. |
| **Mandible** | |
| Swelling | Any swelling of the mandibular region. A “*yes*” was scored if was swelling of the mandibular region. A “*no*” was scored if there was no swelling of the mandibular region. A “*not testable*” was scored if swelling of the mandibular region could not be assessed. |
| Extra-oral laceration | Any extra-oral laceration of the skin of the mandibular region. A “*yes*” was scored if there was any laceration of the mandibular cheek or chin. A “*no*” was scored if there were no laceration of the mandibular cheek or chin. A “*not testable*” was scored if laceration of the mandibular cheek or chin could not be assessed. |
| Jaw movement pain | The presence of pain during opening, protrusion or lateral movement(s) of the mandible. A “*yes*” was scored if there was pain with jaw movement(s). A “*no*” was scored if there was full range movement of the jaw without pain. A “*not testable*” was scored if pain during jaw movements could not be tested, for example when the patient’s state of consciousness hindered  adequate communication. |
| Mouth opening limitations | The reported inability to open the mouth fully or a measured restriction of the mouth opening of 35mm or less during opening of mandible. A “*yes*” was scored in case of mouth opening limitations. A “*no*” was scored if there were no mouth opening limitations. A “*not testable*” was scored if the mouth opening limitations could not be assessed, for example when the patient’s state of consciousness hindered adequate communication. |
| Inferior alveolar nerve paresthesia | Any numbness, change or loss of sensation of the inferior alveolar nerve innervation area (e.g., skin of the lower lip and chin, the mucosa and the gingiva of the inferior vestibule). A “*yes*” was scored if there was inferior alveolar nerve paresthesia. A “*no*” was scored if there was no inferior alveolar nerve paresthesia. A “*not testable*” was scored if the presence of inferior alveolar nerve paresthesia could not be assessed, for example when the patient’s state of consciousness of the patient hindered sufficient communication. |
| Intra-oral hematoma | Any intra-orally localized hematoma either in the mucosa or gingival tissue, including sublingual hematoma. A “*yes*” was scored if there was an intra-oral hematoma. A “*no*” was scored if there was no intra-oral hematoma. A “*not testable*” was scored if the presence of an intra-oral hematoma could not be assessed. |
| Intra-oral laceration | Any intra-orally localized gingival, sublingual or mucosal laceration. A “*yes*” was scored if there was an intra-oral laceration. A “*no*” was scored if there was no intra-oral laceration. A “*not testable*” was scored if the presence of an intra-oral laceration could not be assessed. |
| Palpable step-off | The presence of a palpable bony step-off, step defect or discontinuity in the mandible, with any of any of the following landmarks: (1) alveolar mandibular process intra-orally, (2) inferior mandibular ridge extra-orally and (3) angular, ramus and condylar mandibular process extra-orally. A “*yes*” was scored if there was a palpable step-off. A “*no*” was scored if there was no palpable step-off. A “*not testable*” was scored if the presence of a palpable step-off could not be assessed, for example in cases where swelling and/or hematoma hindered full palpable examination of the mandible. |
| Tooth mobility or avulsion | Mobility or avulsion of any mandibular tooth element (e.g., third (31-38) and fourth (41-48) quadrant tooth elements). A “*yes*” was scored in case of tooth mobility or avulsion. A “*no*” was scored if there was no tooth mobility or avulsion. A “*not testable*” was scored if the presence of tooth mobility or avulsion could not be assessed, for example if the patient could not be suitably instructed. |
| Subjective malocclusion | Misalignment or incorrect relation between the teeth of the two dental arches as experienced by the patient that emerged after the trauma. A “*yes*” was scored if the patient experiences the presence of malocclusion. A “*no*” was scored if the patient did not experience malocclusion. A “*not testable*” was scored if the presence of subjective malocclusion could not be assessed, for example a situation where the patient’s state of consciousness hindered communication. |
| Objective malocclusion | Traumatic misalignment or incorrect relation between the teeth of the two dental arches as objectively identified by the assessor during intra-oral examination assessment. A “*yes*” was scored in there was malocclusion. A “*no*” was scored if there was no malocclusion. A “*not testable*” was scored if the occlusion could not be assessed, for example if the patient had dentures, the patient was edentulous or if the patient was intubated and sedated. |
| Angular compression test pain | Noteworthy presence of pain complaints in the symphyseal or parasymphyseal region induced by simultaneous bilateral pressure of the mandibular angle also known as the angular compression test. A “*yes*” was scored in case of noteworthy presence of pain during the angular compression test. A “*no*“ was scored if there was no pain during the angular compression test. A “*not testable*” was scored when the angular compression test could not be conducted, for example if communication with the patient was not possible. |
| Axial chin pressure test pain | Noteworthy unilateral or bilateral presence of pain complaints of the condylar or temporomandibular region induced by axial pressure on the chin. A “*yes*” was scored in case of noteworthy pain during the axial chin pressure test. A “*no*” was scored if there were no pain during the axial chin pressure test. A “*not testable*” was scored when the axial chin pressure test could not be conducted, for example if communication with the patient was not possible. |
| Tongue blade bite test | The patient’s ability to maintain bilateral intermaxillary fixation of a uniform wooden spatula or tongue depressor (150 x 18 x 1.6 mm) while it was broken by a medial rotation by the assessor. A “*yes*” was scored if the patient was not able to maintain bilateral fixation of the wooden spatula during the tongue blade bite test. A “*no*“ was scored if the patient was able to maintain bilateral fixation of the wooden spatula during the tongue blade bite test. A “*not testable*” was scored if the tongue blade bite test could not be conducted, for example in a situation where the patient could not be instructed. |
